# Efficacy and safety of Xiaoyao San in the treatment of chronic fatigue syndrome: a systematic review and meta-analysis

**DOI:** 10.3389/fphar.2025.1496774

**Published:** 2025-02-06

**Authors:** Qianqian Wang, Jian Zhou, Guanwen Gong

**Affiliations:** ^1^ Department of Preventive Treatment of Diseases, Nantong Hospital of Traditional Chinese Medicine, Nantong, China; ^2^ Department of General Surgery, Affiliated Hospital of Nanjing University of Chinese Medicine, Nanjing, China

**Keywords:** chronic fatigue syndrome, traditional Chinese medicine, Xiaoyao San, standard biomedical treatments, systematic review, meta-analysis

## Abstract

**Background:**

Xiaoyao San (XYS) has been increasingly used in China for treating chronic fatigue syndrome (CFS), but its efficacy and safety remain unclear.

**Objective:**

To systematically evaluate the efficacy and safety of XYS compared to standard biomedical treatments (SBT) in CFS patients.

**Methods:**

A comprehensive search of English and Chinese databases was conducted up to December 2024. Eligible studies included randomized controlled trials comparing XYS or XYS + SBT to SBT alone. Primary outcomes were effective rate (ER) and fatigue scale-14 (FS-14). Secondary outcomes included self-rating anxiety scale (SAS), self-rating depression scale (SDS), and adverse events (AEs). Data were analyzed using Review Manager 5.4, and evidence quality was assessed using the GRADE approach.

**Results:**

Six studies involving 623 patients were included. The meta-analysis showed that XYS-based interventions significantly improved ER (RR = 1.27, 95% CI: 1.18–1.37, I^2^ = 0%) and FS-14 (MD = 1.77, 95% CI: 1.49–2.06, I^2^ = 54%). Subgroup analyses confirmed consistent efficacy for both XYS vs. SBT and XYS + SBT vs. SBT. Anxiety and depression improved significantly in the XYS + SBT group, with SAS (MD = 5.16, 95% CI: 3.84–6.48, I^2^ = 24%) and SDS (MD = 4.62, 95% CI: 3.15–6.09, I^2^ = 0%). Additionally, the risk of AEs was significantly reduced in the XYS + SBT group compared to SBT alone (RR = 0.48, 95% CI: 0.32–0.72, I^2^ = 0%). However, the quality of evidence was rated “low” due to risk of bias and potential publication bias among the studies.

**Conclusion:**

XYS, whether alone or with SBT, is effective and safe for improving ER, fatigue, anxiety, and depression in CFS patients. However, due to the low quality of the evidence, results should be interpreted cautiously. High-quality RCTs with larger sample sizes and longer follow-up are needed to provide stronger evidence for the clinical use of XYS in managing CFS.

**Systematic Review Registration:**

https://www.crd.york.ac.uk/prospero/display_record.php?RecordID=493084, identifier CRD42023493084.

## 1 Introduction

Chronic fatigue syndrome (CFS) is a severe, chronic condition characterized by debilitating fatigue, post-exertional malaise, and cognitive impairments (Grach et al., 2023; McKeever, 2024). This persistent fatigue lasts or recurs for more than 6 months and is not relieved by rest (Fukuda et al., 1994). Mental health issues are prevalent among CFS patients, with depression, anxiety disorders, and insomnia being common comorbidities (Leong et al., 2022; König et al., 2024). These symptoms significantly disrupt patients’ daily lives. Individuals with higher levels of education, women, and those over 50 years old are more likely to develop CFS(Liu et al., 2023a). The prevalence of CFS is estimated at 0.89% globally (Lim et al., 2020), ranging from 0.1% to 2.2% in Europe (Estévez-López et al., 2020), and as high as 12.54% in China (Wu et al., 2020). Due to the absence of specific diagnostic markers, prevalence rates vary widely, and it is speculated that many CFS cases remain undiagnosed (Araja et al., 2021). Viral infections are considered potential triggers for the disease (Chang et al., 2023; Seton et al., 2024). Notably, the global prevalence of CFS among long COVID-19 patients is reported to be as high as 45.2% (Salari et al., 2022). Given the significant overlap with post-COVID syndrome, CFS has garnered unprecedented attention in the medical community in recent years (Davis et al., 2023).

The pathogenesis of CFS remains unclear, involving complex disturbances across immunological, metabolic, gastrointestinal, neurological, and neuroendocrine systems (Seton et al., 2024). Management of CFS primarily focuses on alleviating common symptoms and includes non-pharmacological treatments such as pacing (Grach et al., 2023), graded exercise therapy (Gaunt et al., 2024), and cognitive behavioral therapy (Kuut et al., 2024), alongside pharmacological interventions like antidepressants, sleep aids, supplements, and vitamin deficiency treatment (Grach et al., 2023). However, only about 4% of CFS patients report steady improvement in their condition (Chu et al., 2019). Currently, the U.S. Food and Drug Administration has not approved any specific treatments for CFS (Dukes et al., 2021). It is estimated that 25.7% of CFS patients are severely affected to the point of being unable to work, with 16.2% of these individuals bedridden and requiring nursing care (Conroy et al., 2021). The high cost of care places a substantial financial burden on both individuals and society (Zhao T. et al., 2023). Therefore, there is an urgent need to explore effective treatment methods for CFS.

Xiaoyao San (XYS), a classical Chinese herbal prescription originating from the Taiping Huimin Hejiju Fang (Chen et al., 2022), is considered a promising alternative therapy for CFS. In traditional Chinese medicine (TCM), CFS is categorized under “consumptive disease,” a condition first documented in the Jin Kui Yao Lue. The basic TCM pathogenesis of CFS includes “liver qi stagnation,” “spleen deficiency,” and “deficiency of both qi and blood” (Luo et al., 2008). XYS functions to soothe the liver, strengthen the spleen, and replenish qi and blood. Research has shown that XYS effectively mitigates depressive symptoms, alleviates anxiety and tension, and produces fewer adverse effects than conventional antidepressants (Chen et al., 2022; Tong et al., 2023). Furthermore, the tablet form of XYS has been registered in the European Union, where it is utilized as a self-care option to help European patients alleviate mental stress and exhaustion (Wang et al., 2022). Building on these benefits, TCM doctors in China typically adjust the ingredients and dosage of XYS based on a patient’s chief complaints and primary symptoms to further optimize therapeutic effects.

This is the first systematic review and meta-analysis on evaluating the efficacy and safety of XYS in treating CFS. By evaluating the existing evidence, this study aims to fill the existing research gaps, provide references for clinicians and future research on CFS management.

## 2 Methods

This study was conducted in accordance with the Preferred Reporting Items for Systematic Reviews and Meta-Analyses (PRISMA) guidelines and has been registered in PROSPERO (Identifier: CRD42023493084).

### 2.1 Search strategy

Four English and four Chinese databases were searched using a pre-designed strategy from inception to December 2024, including PubMed, EMBASE, Cochrane Library, Web of Science, Chinese National Knowledge Infrastructure, Chinese Scientific Journals Database, Wanfang Data, and Chinese Biomedicine Literature Database. The search terms included “Fatigue Syndrome, Chronic,” “chronic fatigue syndrome,” “CFS,” “Chronic Fatigue Disorder,” “Fatigue Disorder, Chronic,” “Myalgic Encephalomyelitis,” “ME,” “Encephalomyelitis, Myalgic,” “Systemic Exertion Intolerance Disease,” “Chronic Fatigue and Immune Dysfunction Syndrome,” “xiaoyaosan,” “xiaoyao*,” “Xiao Yao*,” “Randomized controlled trial,” “Randomized.” The detailed search strategy is provided in the supplemental file. Additionally, relevant systematic reviews were manually searched to identify studies eligible for inclusion. The search was conducted in both English and Chinese.

### 2.2 Study selection criteria

#### 2.2.1 Types of studies

Randomized controlled trials (RCTs).

#### 2.2.2 Types of participants

Participants diagnosed with CFS by the Center for Disease Control (CDC) criteria (1994, 2001, or 2005) were eligible for inclusion, with no restrictions on age, gender, or race.

#### 2.2.3 Types of interventions

The experimental group received XYS treatment, or combination of XYS and standard biological treatment (SBT). The control group received SBT. The route of administration was not limited, with no restrictions on dosage or treatment duration. The botanical composition of XYS was as follows (Ha et al., 2023): the dried root of Bupleurum chinese DC [Apiaceae; Bupleuri radix]; the dried root of Angelica sinensis (Oliv.) Diels [Apiaceae; Angelicae sinensis radix]; the dried root Paeonia lactiflora Pall [Paeoniaceae; Paeoniae radix alba]; the dried rhizome of Atractylodes macrocephala Koidz [Compositae; Atractylodis macrocephalae rhizoma]; the dried sclerotium of Poria cocos (Schw.) Wolf [Polyporaceae; Poria cocos]; the dried rhizome of Zingiber officinale Roscoe [Zingiberaceae; Zingiberis rhizoma recens]; the dried aerial parts of Mentha canadensis L [Lamiaceae; Menthae herba]; the dried root and rhizome of Glycyrrhiza uralensis Fisch [Leguminosae; Glycyrrhizae radix et rhizoma]. Modified XYS (MXYS) is based on the core formula of XYS but incorporates additional botanical drugs to address specific clinical presentations. For instance, in cases of heat signs, the dried root bark of Paeonia × suffruticosa Andrews [Paeoniaceae; Moutan cortex] and the dried ripe fruit of Gardenia jasminoides J. Ellis [Rubiaceae; Gardeniae fructus] were added. For qi deficiency, the dried root of Codonopsis pilosula (Franch.) Nannf. [Campanulaceae; Codonopsis radix] and the dried root of Dioscorea opposita Thunb. [Dioscoreaceae; Dioscoreae rhizoma] were added. By retaining the base composition of XYS and tailoring it with specific botanical drugs for symptom management, MXYS remains classified under the broader XYS formulation family.

#### 2.2.4 Types of comparators

This review included the following comparative studies: XYS vs. SBT, XYS + SBT vs. SBT.

#### 2.2.5 Types of outcome measures

Primary outcomes: effective rate (ER), fatigue scale-14 (FS-14). Secondary outcomes: self-rating anxiety scale (SAS), self-rating depression scale (SDS), and adverse events (AEs). In this study, ER was used to compare the therapeutic effects between the experimental and control groups on CFS. ER reflects the proportion of patients who experienced significant symptom improvement following the intervention.

### 2.3 Data extraction

Two reviewers (QW and JZ) independently screened titles and abstracts, and subsequently reviewed the full texts to identify eligible trials. They then extracted data using a pre-designed form. In cases where study data required clarification, the authors were contacted. Data extraction included: 1) Basic information (first author, country, year of publication); 2) Primary baseline characteristics (sample size, male-to-female ratio, age, course of disease); 3) Details of interventions in the experimental and control groups; 4) Key elements for assessing risk of bias; and 5) Outcome indicators of interest. In instances where the two reviewers disagreed on data extraction, a third reviewer (GG) acted as an arbitrator.

### 2.4 Risk of bias assessment

The following seven bias domains were independently evaluated by two reviewers (QW and JZ) using the Cochrane Handbook for Systematic Reviews of Interventions (Cumpston et al., 2019): random sequence generation, allocation concealment, blinding of participants and personnel, blinding of outcome assessment, incomplete outcome data, selective reporting, and other biases. The risk of bias for each domain was categorized as low, high, or unclear. In cases of disagreement between the two reviewers, a third reviewer (GG) served as an arbitrator.

### 2.5 Data synthesis and analysis

We assessed dichotomous outcomes, including ER and AEs, using relative risk (RR) with 95% confidence intervals (CIs). For continuous outcomes, such as FS-14, SAS, and SDS, we applied mean difference (MD) with 95% CIs. Heterogeneity was evaluated using the χ^2^ test and the I^2^ statistic. A fixed-effects model was employed when the p-value was > 0.10 or I^2^ ≤ 50%, while a random-effects model was used when the p-value was ≤ 0.10 or I^2^ > 50%. When heterogeneity was high, subgroup analysis and sensitivity analysis were performed. Potential publication bias was assessed using a funnel plot when more than 10 studies were included. For fewer than 10 studies, Egger’s test was used to detect publication bias. If potential bias was identified, additional sensitivity analyses or the trim-and-fill method were performed to assess its impact on the results. Data analysis was performed using Review Manager 5.4, and the overall quality of evidence was evaluated using the Grading of Recommendations, Assessment, Development, and Evaluation (GRADE) approach.

## 3 Results

**FIGURE 1:**
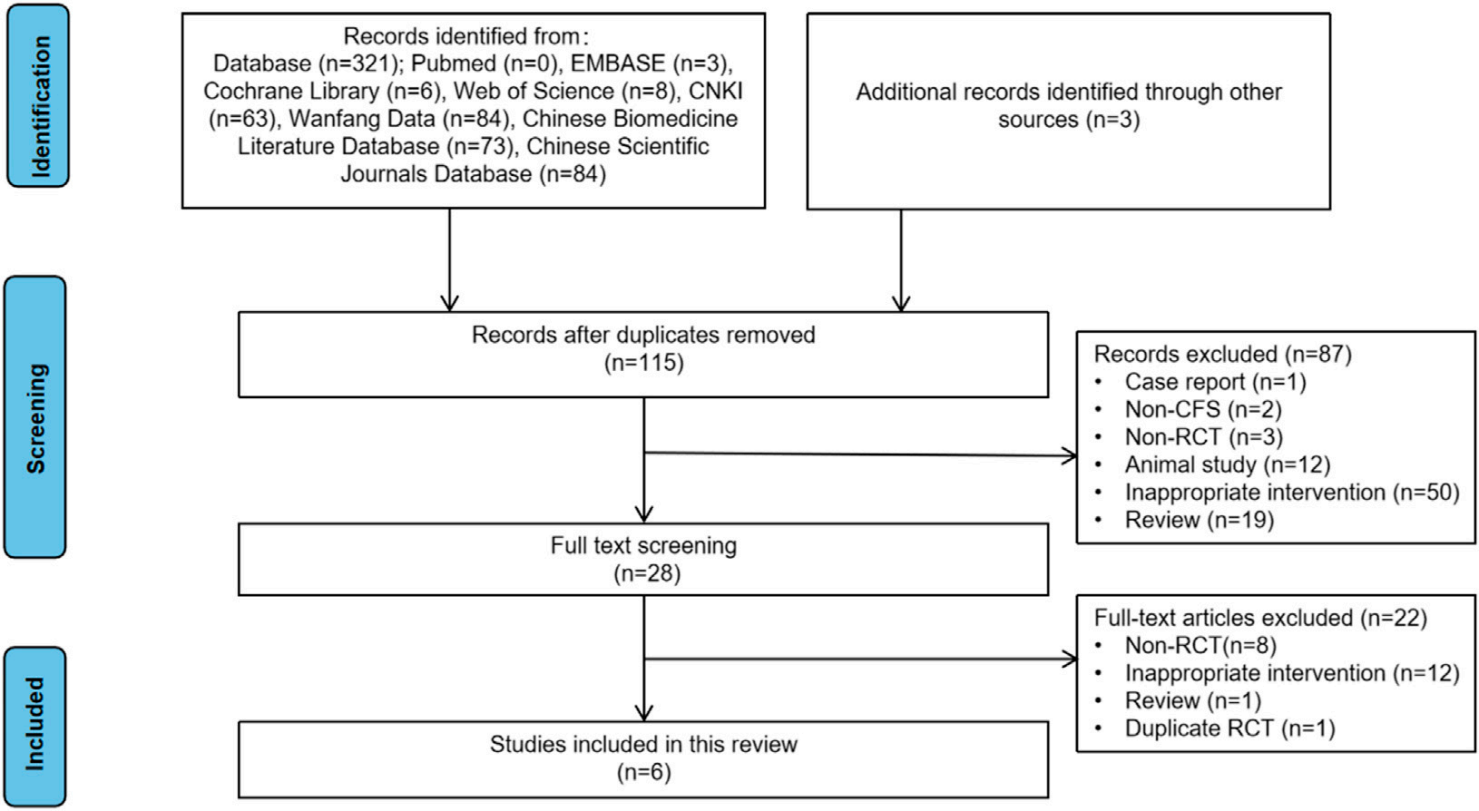
Flowchart of study selection.

Through a comprehensive search of both Chinese and English databases, 324 studies were initially identified. After removing 209 duplicate records, 115 studies remained. Screening of abstracts led to the exclusion of 87 studies that did not meet the inclusion criteria. Full-text reviews were conducted for the remaining 28 studies, resulting in the exclusion of 8 non-RCTs, 12 RCTs with inappropriate interventions, 1 duplicate RCT, and 1 review article. Ultimately, 6 RCTs were included in the final analysis (Figure 1).

### 3.1 Study characteristics

Six RCTs (Jie and Wang, 2009; Qin et al., 2013; Guo and Guo, 2015; Xu, 2015; Shi, 2019; Chen, 2021) involving 623 participants were included in this study (Table 1). All studies were conducted in China between 2009 and 2021. For the study design, all six were two-arm, parallel-design RCTs. Among the six RCTs, two (Shi, 2019; Chen, 2021) compared XYS to SBT, four (Jie and Wang, 2009; Qin et al., 2013; Guo and Guo, 2015; Xu, 2015) compared combination of XYS and SBT to SBT alone. Regarding the dosage form and route administration of XYS, three studies (Jie and Wang, 2009; Guo and Guo, 2015; Xu, 2015) used oral tablets, two studies (Shi, 2019; Chen, 2021) used oral decoctions, and one study (Qin et al., 2013) used a powder applied to the navel (Table 2). For the type of SBT, three studies (Jie and Wang, 2009; Guo and Guo, 2015; Xu, 2015) used paroxetine, one study (Qin et al., 2013) used oryzanol, one study (Shi, 2019) used oryzanol and vtamin B, one study (Chen, 2021) used oryzanol, vitamin B1 and adenosine triphosphate. Sample sizes ranged from 60 to 180 participants. The treatment durations ranging from 21 to 60 days. As for the diagnostic criteria for CFS, five studies (Jie and Wang, 2009; Qin et al., 2013; Xu, 2015; Shi, 2019; Chen, 2021) used the CDC-1994 criteria and one study (Guo and Guo, 2015) used the CDC-2001 criteria. As for the TCM syndrome types for CFS, four studies (Qin et al., 2013; Guo and Guo, 2015; Shi, 2019; Chen, 2021) used liver depression and spleen deficiency syndrome.

**TABLE 1:** Study characteristics.

Study ID	Country	Diagnostic criteria/TCM syndrome type	Sample size (E/C)	Male to female ratio (E/C)	Age (years) (mean ± sd) (E/C)	Course of disease (months) (E/C)	Experimental group intervention	Control group intervention	Duration (days)	Outcome measures/Measurement method	Adverse events
Chen (2021)	China	CDC- 1994 criteria/Liver depression and spleen deficiency syndrome	63 (33/30)	15:18/14:16	33.8 ± 13.1/33.6 ± 13.2	18.51 ± 9.03/16.32 ± 8.94	XYS (bid)	Oryzanol (20 mg, tid)+ Vitamin B1 (20 mg, tid)+ Adenosine triphosphate (20 mg, tid)	60	①②⑤	0/0
Shi (2019)	China	CDC- 1994 criteria/Liver depression and spleen deficiency syndrome	160 (78/82)	35:43/32:50	41.51 ± 9.347/40.55 ± 9.775	10.8–36/8.4–24	MXYS (bid)	Oryzanol (20 mg, tid)+ Vitamin B (2 pills, tid)	21	①②⑤	0/0
Guo and Guo (2015)	China	CDC- 2001 criteria/Liver depression and spleen deficiency syndrome	90 (45/45)	19:26/20:25	38.27 ± 6.54/39.20 ± 6.85	18.26 ± 5.54/17.77 ± 6.23	MXYS (tid) + Paroxetine (10–30 mg, qd)	Paroxetine (10–30 mg, qd)	56	①②③④⑤	7/15
Qin et al. (2013)	China	CDC- 1994 criteria/Liver depression and spleen deficiency syndrome	60 (30/30)	8:22/10:20	36.5 ± 0.9/37.2 ± 0.5	22 ± 1/23 ± 1	MXYS (qd) + Oryzanol (20 mg, tid)	Oryzanol (20 mg, tid)	56	①②⑤	2/0
Jie and Wang (2009)	China	CDC- 1994 criteria/NR	70 (35/35)	14:21/16:19	34.55 ± 7.45/33.95 ± 6.54	14.50 ± 4.50/14.20 ± 4.15	XYS (tid) + Paroxetine (20–40 mg/d)	Paroxetine (20–40 mg/d)	56	①②③④⑤	3/10
Xu (2015)	China	CDC- 1994 criteria/NR	180 (90/90)	38:52/40:50	33.98 ± 3.67/34.61 ± 3.54	NR	MXYS (tid) + Paroxetine (10–30 mg, qd)	Paroxetine (10–30 mg, qd)	56	①②③④⑤	14/30

Abbreviations: CFS, chronic fatigue syndrome; SBT, standard biomedical treatment; XYS, Xiaoyao San; MXYS, Modified Xiaoyao San; CDC, the Center for Disease Control; NR, not reported, ①effective rate, ②fatigue, ③anxiety, ④depression, ⑤adverse events.

**TABLE 2:** Herbal prescription and its components in the included RCTs.

Study ID	Prescription (volume per intake, frequency,administration route); extraction process	Ingredients of Xiaoyao San	Medical institution	Quality control	Chemical profile
Chen (2021)	Xiaoyao San; (NR, bid, oral); decoction from mixtures of botanical drugs	Dried root of Bupleurum chinese DC [Apiaceae; Bupleuri radix] 6 g, Dried root of Angelica sinensis (Oliv.) Diels [Apiaceae; Angelicae sinensis radix] 10 g, Dried root Paeonia lactiflora Pall [Paeoniaceae; Paeoniae radix alba] 12 g, Dried rhizome of Atractylodes macrocephala Koidz [Compositae; Atractylodis macrocephalae rhizoma] 12 g, Dried sclerotium of Poria cocos (Schw.) Wolf [Polyporaceae; Poria cocos] 12 g, Dried rhizome of Zingiber officinale Roscoe [Zingiberaceae; Zingiberis rhizoma recens] 3 g, Dried aerial parts of Mentha canadensis L [Lamiaceae; Menthae herba] 5 g, Dried root and rhizome of Glycyrrhiza uralensis Fisch [Leguminosae; Glycyrrhizae radix et rhizoma] 6 g	Hengxian People’s Hospital, Nanning, China	NR	NR
Shi (2019)	Modified Xiaoyao San; (300 mL, bid, oral); decoction from mixtures of botanical drugs	Dried root of Bupleurum chinese DC [Apiaceae; Bupleuri radix] 6 g, Dried root of Angelica sinensis (Oliv.) Diels [Apiaceae; Angelicae sinensis radix] 10 g, Dried root Paeonia lactiflora Pall [Paeoniaceae; Paeoniae radix alba] 10 g, Dried rhizome of Atractylodes macrocephala Koidz [Compositae; Atractylodis macrocephalae rhizoma] 10 g, Dried sclerotium of Poria cocos (Schw.) Wolf [Polyporaceae; Poria cocos] 20 g, Dried aerial parts of Mentha canadensis L [Lamiaceae; Menthae herba] 6 g, Dried root and rhizome of Glycyrrhiza uralensis Fisch [Leguminosae; Glycyrrhizae radix et rhizoma] 6 g, Dried root of Codonopsis pilosula (Franch.) Nannf. [Campanulaceae; Codonopsis radix] 15 g, Dried root of Dioscorea opposita Thunb. [Dioscoreaceae; Dioscoreae rhizoma] 20 g	Beijing Fangshan District Traditional Chinese Medicine Hospital, Beijing, China	NR	NR
Guo and Guo (2015)	Modified Xiaoyao San; (4 pills, tid, oral); Add water to the medicine and boil it. Filter, concentrate to a suitable concentration, dry, add auxiliary materials for granulation, and press into tablets	The Danzhi Xiaoyao Tablet was produced based on the procedure described in the Drug Standards of the Ministry of Health of the People’s Republic of China for Chinese Medicine Formulas (Pharmacopoeia Committee of the Ministry of Health of the People’s Republic of China, 1995). Dried root of Bupleurum chinese DC [Apiaceae; Bupleuri radix] 180 g, Dried root of Angelica sinensis (Oliv.) Diels [Apiaceae; Angelicae sinensis radix] 180 g, dried root Paeonia lactiflora Pall [Paeoniaceae; Paeoniae radix alba] 180 g, Dried rhizome of Atractylodes macrocephala Koidz [Compositae; Atractylodis macrocephalae rhizoma] 180 g, Dried sclerotium of Poria cocos (Schw.) Wolf [Polyporaceae; Poria cocos] 180 g, Dried root and rhizome of Glycyrrhiza uralensis Fisch [Leguminosae; Glycyrrhizae radix et rhizoma] 90 g, Dried ripe fruit of Gardenia jasminoides J. Ellis [Rubiaceae; Gardeniae fructus] 90 g, Dried root bark of Paeonia × suffruticosa Andrews [Paeoniaceae; Moutan cortex] 90 g. Each 0.35 g of the raw botanical drugs yielded one pill	Henan University of Chinese Medicine, Zhengzhou, China	Drug Standards of the Ministry of Health of the People’s Republic of China for Chinese Medicine Formulas (Pharmacopoeia Committee of the Ministry of Health of the People’s Republic of China, 1995)	NR
Qin et al. (2013)	Modified Xiaoyao San; (10 g, qd, topical); powder from mixtures of botanical drugs	Dried root of Bupleurum chinese DC [Apiaceae; Bupleuri radix] 50 g, Dried root of Angelica sinensis (Oliv.) Diels [Apiaceae; Angelicae sinensis radix] 50 g,Dried root Paeonia lactiflora Pall [Paeoniaceae; Paeoniae radix alba] 50 g, Dried rhizome of Atractylodes macrocephala Koidz [Compositae; Atractylodis macrocephalae rhizoma] 50 g, Dried sclerotium of Poria cocos (Schw.) Wolf [Polyporaceae; Poria cocos] 50 g, Dried rhizome of Zingiber officinale Roscoe [Zingiberaceae; Zingiberis rhizoma recens] 20 g, Dried aerial parts of Mentha canadensis L [Lamiaceae; Menthae herba] 20 g, Dried root and rhizome of Glycyrrhiza uralensis Fisch [Leguminosae; Glycyrrhizae radix et rhizoma] 20 g, Dried ripe fruit of Gardenia jasminoides J. Ellis [Rubiaceae; Gardeniae fructus] 20 g, Dried root bark of Paeonia × suffruticosa Andrews [Paeoniaceae; Moutan cortex] 20 g, Dried root of Codonopsis pilosula (Franch.) Nannf. [Campanulaceae; Codonopsis radix] 50 g	Xianxian Tospital of Traditional Chinese Medicine, Hebei, China	NR	NR
Jie and Wang (2009)	Xiaoyao San; (8 pills, tid, oral); Crush the botanical drugs into fine powder, sieve, and mix evenly. Add 135–145 g of refined honey to every 100 g of powder to form small or large honeyed pills	The Xiaoyao Pill was produced based on the procedure described in the Chinese Pharmacopoeia 2020 Edition (National Pharmacopoeia Commission, 2020). Dried root of Bupleurum chinese DC [Apiaceae; Bupleuri radix] 100 g, Dried root of Angelica sinensis (Oliv.) Diels [Apiaceae; Angelicae sinensis radix] 100 g, Dried root Paeonia lactiflora Pall [Paeoniaceae; Paeoniae radix alba] 100 g, Dried rhizome of Atractylodes macrocephala Koidz [Compositae; Atractylodis macrocephalae rhizoma] 100 g, Dried sclerotium of Poria cocos (Schw.) Wolf [Polyporaceae; Poria cocos] 100 g, Dried rhizome of Zingiber officinale Roscoe [Zingiberaceae; Zingiberis rhizoma recens] 100 g, Dried aerial parts of Mentha canadensis L [Lamiaceae; Menthae herba] 20 g, Dried root and rhizome of Glycyrrhiza uralensis Fisch [Leguminosae; Glycyrrhizae radix et rhizoma] 80 g. Each 3.0 g of the raw botanical drugs yielded eight pills	Tianshui Psychiatric Hospital, Tianshui, China	Chinese Pharmacopoeia (National Pharmacopoeia Commission, 2020)	NR
Xu (2015)	Modified Xiaoyao San; (4 pills, tid, oral); Add water to the medicine and boil it. Filter, concentrate to a suitable concentration, dry, add auxiliary materials for granulation, and press into tablets	The Danzhi Xiaoyao Tablet was produced based on the procedure described in the Drug Standards of the Ministry of Health of the People’s Republic of China for Chinese Medicine Formulas (Pharmacopoeia Committee of the Ministry of Health of the People’s Republic of China, 1995). Dried root of Bupleurum chinese DC [Apiaceae; Bupleuri radix] 180 g, Dried root of Angelica sinensis (Oliv.) Diels [Apiaceae; Angelicae sinensis radix] 180 g, dried root Paeonia lactiflora Pall [Paeoniaceae; Paeoniae radix alba] 180 g, Dried rhizome of Atractylodes macrocephala Koidz [Compositae; Atractylodis macrocephalae rhizoma] 180 g, Dried sclerotium of Poria cocos (Schw.) Wolf [Polyporaceae; Poria cocos] 180 g, Dried root and rhizome of Glycyrrhiza uralensis Fisch [Leguminosae; Glycyrrhizae radix et rhizoma] 90 g, Dried ripe fruit of Gardenia jasminoides J. Ellis [Rubiaceae; Gardeniae fructus] 90 g, Dried root bark of Paeonia × suffruticosa Andrews [Paeoniaceae; Moutan cortex] 90 g. Each 0.35 g of the raw botanical drugs yielded one pill	Liuzhi Special District People’s Hospital, Guizhou, China	Drug Standards of the Ministry of Health of the People’s Republic of China for Chinese Medicine Formulas (Pharmacopoeia Committee of the Ministry of Health of the People’s Republic of China, 1995)	NR

### 3.2 Risk of bias

**FIGURE 2:**
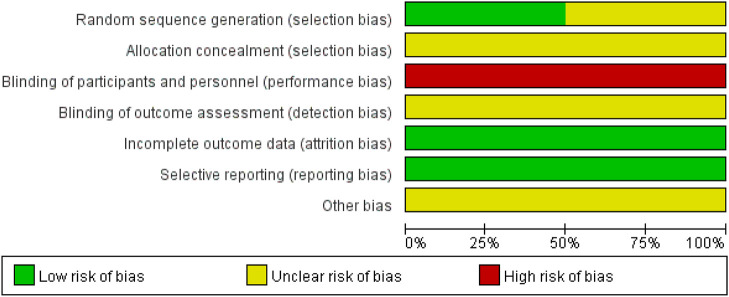
Summary of the risk of bias for each included study.

**FIGURE 3:**
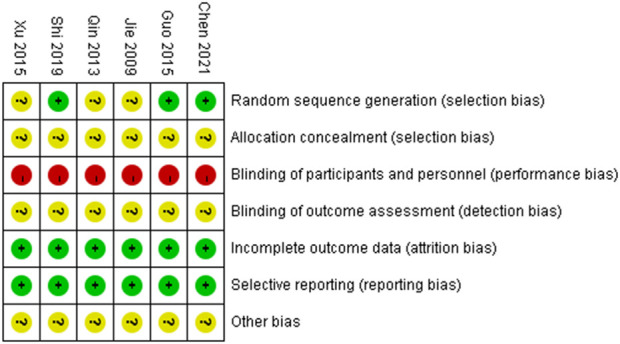
Graph showing the risk of bias across all included studies.

The risk of bias is illustrated in Figures 2, 3.

#### 3.2.1 Random sequence generation

Three studies (Guo and Guo, 2015; Shi, 2019; Chen, 2021) used a random number table and were assessed as having a low risk of bias. The remaining three studies did not describe the method for random sequence generation and were therefore rated as having an unclear risk of bias.

#### 3.2.2 Allocation concealment

All six studies did not report the methods used for allocation concealment and were thus rated as having an unclear risk of bias.

#### 3.2.3 Blinding of participants and personnel

In all six studies, there were clearly different appearance between XYS and SBT, making it impossible to blind participants and personnel to group allocation. No blinding methods were implemented, and all studies were therefore assessed as having a high risk of bias.

#### 3.2.4 Blinding of outcome assessment

None of the six studies provided an explanation regarding the blinding of outcome assessors. As a result, all studies were rated as having an unclear risk of bias.

#### 3.2.5 Incomplete outcome data

All six studies reported complete outcome data and were assessed as having a low risk of bias.

#### 3.2.6 Selective reporting

All six studies reported the outcomes that were mentioned in their respective methods sections and were therefore assessed as having a low risk of bias.

#### 3.2.7 Other bias

Due to insufficient evidence to identify other forms of bias, all six studies were considered to have an unclear risk of bias in this category.

### 3.3 Outcomes

#### 3.3.1 ER

**FIGURE 4:**
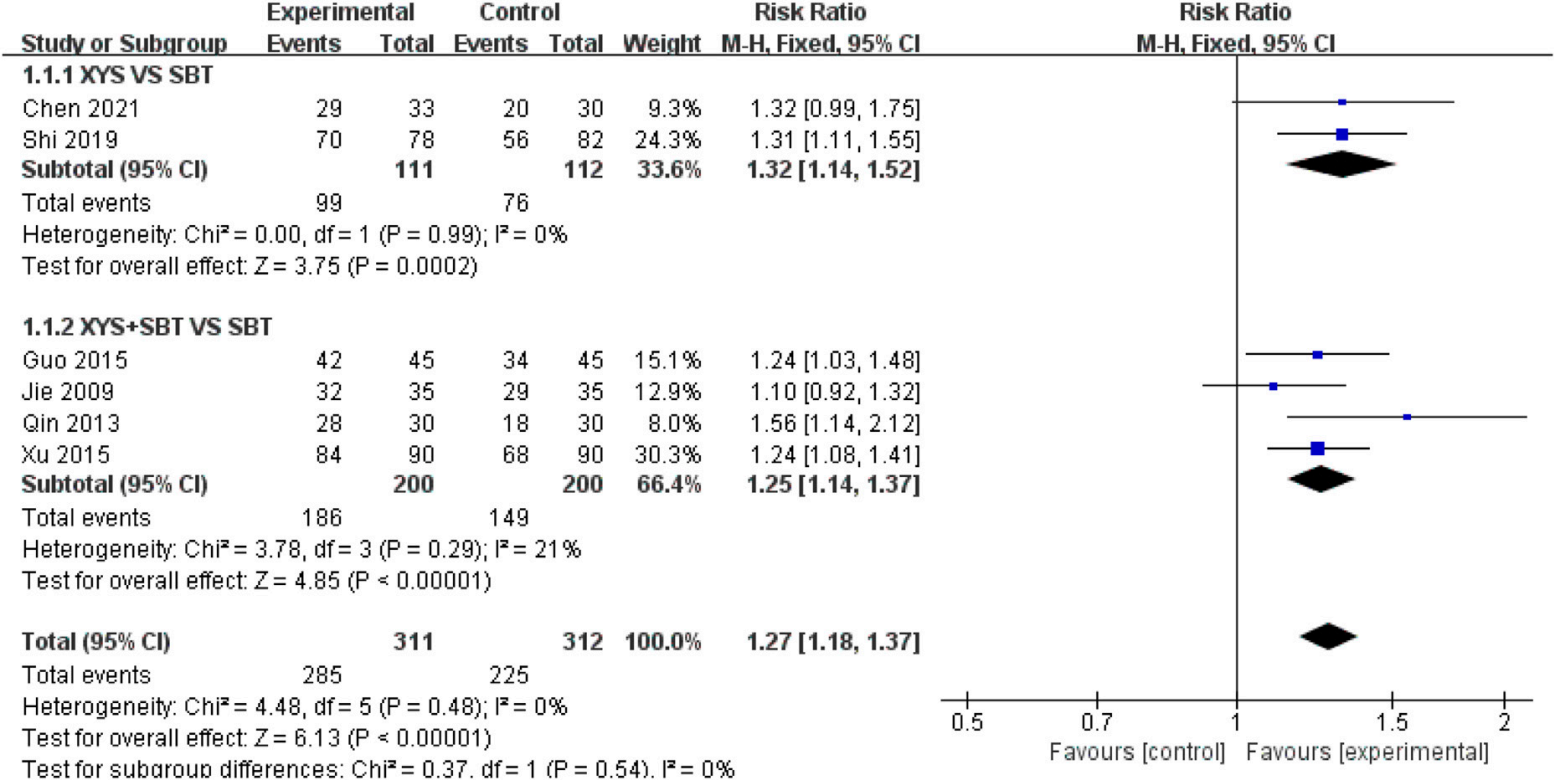
Forest plot of the comparison for effective rate (ER) between the XYS and SBT groups.

A total of six studies involving 623 CFS participants (Jie and Wang, 2009; Qin et al., 2013; Guo and Guo, 2015; Xu, 2015; Shi, 2019; Chen, 2021) were included in the meta-analysis to assess the ER. The pooled analysis demonstrated that the experimental group had a significantly higher ER compared to the control group (RR = 1.27; 95% CI: 1.18–1.37; p < 0.00001). No heterogeneity was observed (I^2^ = 0%; p = 0.48). Subgroup analyses were conducted to explore the effects of different intervention types: 1) XYS vs. SBT: Two studies involving 223 participants (Shi, 2019; Chen, 2021) reported a significantly higher ER in the XYS group compared to the SBT group (RR = 1.32; 95% CI: 1.14–1.52; p = 0.0002), with no heterogeneity (I^2^ = 0%; p = 0.99). 2) XYS + SBT vs. SBT: Four studies involving 400 participants (Jie and Wang, 2009; Qin et al., 2013; Guo and Guo, 2015; Xu, 2015) showed that the combination of XYS and SBT significantly improved ER compared to SBT alone (RR = 1.25; 95% CI: 1.14–1.37; p < 0.00001), with low heterogeneity (I^2^ = 21%; p = 0.29) (Figure 4).

#### 3.3.2 FS-14

**FIGURE 5:**
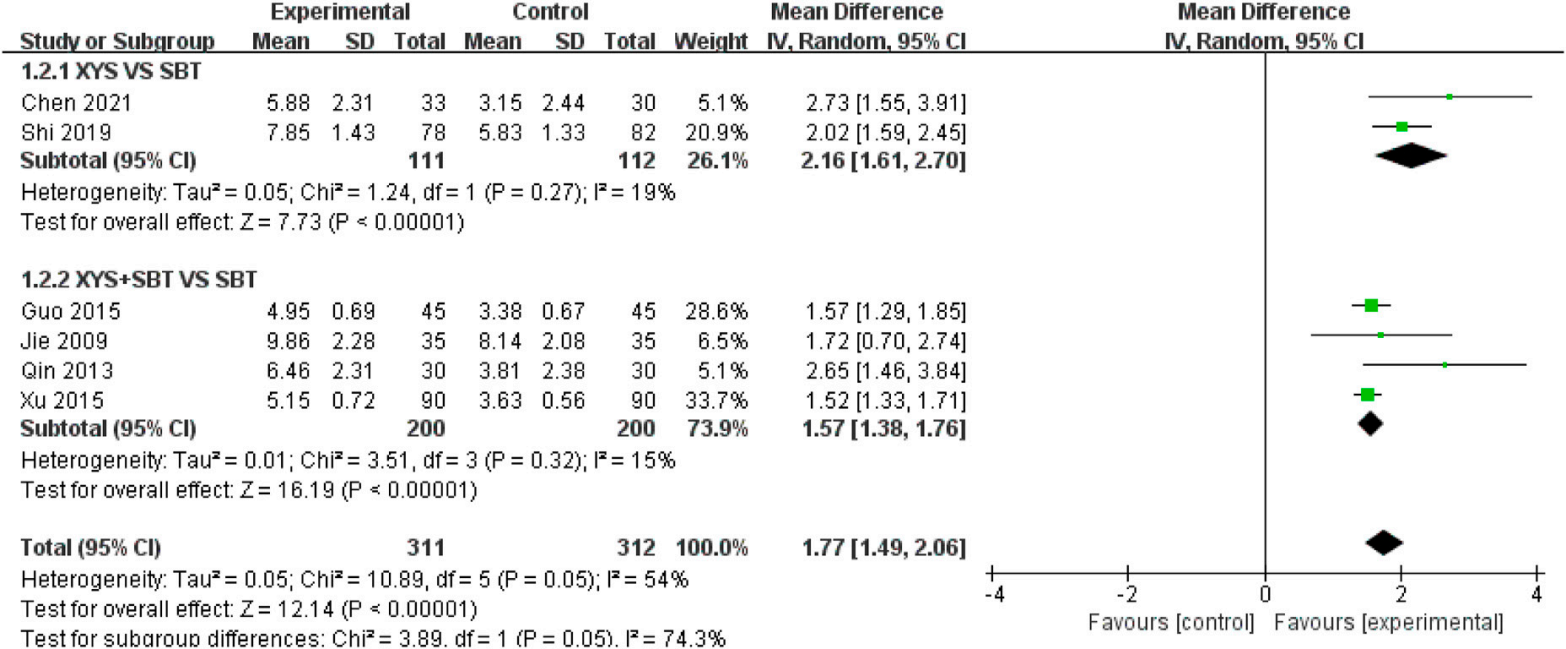
Forest plot of the comparison for fatigue scale-14(FS-14) between the XYS and SBT groups.

A total of six studies involving 623 participants (Jie and Wang, 2009; Qin et al., 2013; Guo and Guo, 2015; Xu, 2015; Shi, 2019; Chen, 2021) were included in the meta-analysis to evaluate the effect of interventions on fatigue, as measured by FS-14. The pooled analysis demonstrated a significant improvement in fatigue symptoms in the experimental group compared to the control group (MD = 1.77; 95% CI: 1.49–2.06; p < 0.00001). Moderate heterogeneity was observed (I^2^ = 54%; p = 0.05). Subgroup analyses were performed to explore the effects of different intervention types and sources of heterogeneity: 1) XYS vs. SBT: Two studies involving 223 participants (Shi, 2019; Chen, 2021) showed that XYS significantly improved fatigue symptoms compared to SBT (MD = 2.16; 95% CI: 1.61–2.70; p < 0.00001), with low heterogeneity (I^2^ = 19%; p = 0.27). 2) XYS + SBT vs. SBT: Four studies involving 400 participants (Jie and Wang, 2009; Qin et al., 2013; Guo and Guo, 2015; Xu, 2015) demonstrated that the combination of XYS and SBT significantly improved fatigue symptoms compared to SBT alone (MD = 1.57; 95% CI: 1.38–1.76; p < 0.00001), with low heterogeneity (I^2^ = 15%; p = 0.32) (Figure 5). The heterogeneity was reduced after subgroup analysis, and the heterogeneity came from intervention types. To assess the robustness of the results, a sensitivity analysis was conducted by sequentially omitting each study. The pooled effect estimates remained consistent, demonstrating the stability of the meta-analysis results.

#### 3.3.3 SAS

**FIGURE 6:**
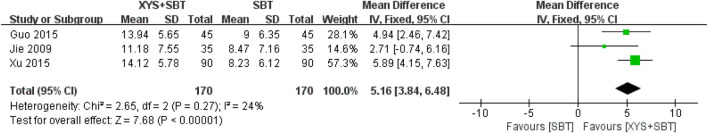
Forest plot of the comparison for self-rating anxiety scale (SAS) between the XYS + SBT and SBT groups.

A total of three studies involving 340 participants (Jie and Wang, 2009; Guo and Guo, 2015; Xu, 2015) were included in the meta-analysis to assess the effect of combination of XYS and SBT on anxiety symptoms, as measured by the SAS. The pooled analysis demonstrated a significant improvement in anxiety symptoms in the combination group compared to the SBT group (MD = 5.16; 95% CI:3.84–6.48; p < 0.00001). Low heterogeneity was observed among the studies (I^2^ = 24%; p = 0.27) (Figure 6).

#### 3.3.4 SDS

**FIGURE 7:**
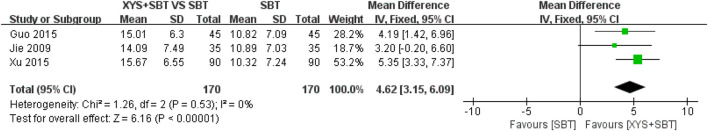
Forest plot of the comparison for self-rating depression scale (SDS) between the XYS + SBT and SBT groups.

A total of three studies involving 340 participants (Jie and Wang, 2009; Guo and Guo, 2015; Xu, 2015) were included in the meta-analysis to assess the effect of combination of XYS and SBT on depression symptoms, as measured by the SDS. The pooled analysis demonstrated a significant improvement in depression symptoms in the combination group compared to the SBT group (MD = 4.62; 95% CI:3.15–6.09; p < 0.00001). No heterogeneity was observed among the studies (I^2^ = 0%; p = 0.53) (Figure 7).

#### 3.3.5 AEs

Six studies reported AEs. Two studies (Shi, 2019; Chen, 2021) indicated no occurrence of AEs. One study (Xu, 2015) reported nausea, dry mouth, headache, and dizziness in the XYS + paroxetine group, while the paroxetine group experienced somnolence, dizziness, dry mouth, and nausea. Similarly, another study (Guo and Guo, 2015) documented nausea, dizziness, and dry mouth in the XYS + paroxetine group, with the paroxetine group reporting similar events. One study (Jie and Wang, 2009) observed nausea and dry mouth in both groups. External application of XYS in one study (Qin et al., 2013) led to redness of the skin around the umbilicus, attributed to the stimulation of the dried rhizome of Zingiber officinale Roscoe [Zingiberaceae; Zingiberis rhizoma recens].

**FIGURE 8:**
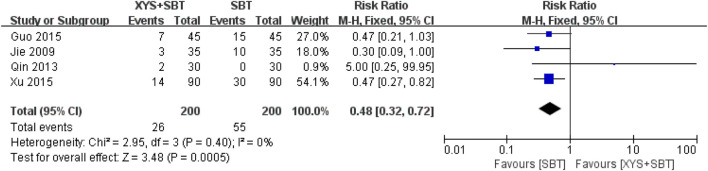
Forest plot of the comparison for adverse events (AEs) between the XYS + SBT and SBT groups.

Four studies involving 400 CFS patients (Jie and Wang, 2009; Qin et al., 2013; Guo and Guo, 2015; Xu, 2015) demonstrated a significantly lower risk of AEs in the XYS + SBT group compared to the SBT group (RR = 0.48; 95% CI: 0.32–0.72; p = 0.0005). No heterogeneity was observed across the included studies (I^2^ = 0%; p = 0.40) (Figure 8). The overall incidence of AEs was 13% in the XYS + SBT group and 27.5% in the SBT group. The forest plot (Figure 8) supports the consistent findings of reduced AEs in the combination therapy group.

### 3.4 Publication bias

**FIGURE 9:**
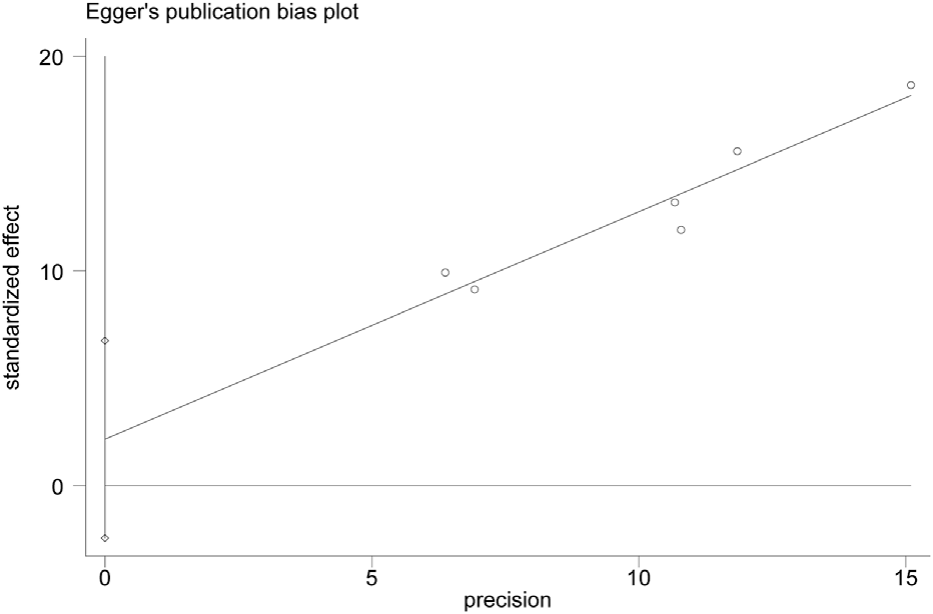
Egger’s publication plot of effective rate (ER).

As only six studies were included, a funnel plot could not be constructed to assess publication bias. Egger’s test of ER showed a bias coefficient of 2.15 (95% CI: -2.45 to 6.75, p = 0.264), indicating no significant publication bias. (Figure 9).

### 3.5 Quality of evidence

The quality of evidence for XYS and combination therapies in treating CFS was rated as “low” for outcomes including ER, FS-14, SAS, SDS, and AEs (Table 3). This low rating primarily resulted from several methodological limitations. First, half of the included studies did not report their randomization methods, and none provided detailed information on allocation concealment or blinding of outcome assessment. The visual differences between XYS and SBT further prevented the implementation of double-blinding in all studies, contributing to a serious risk of bias. Second, although Egger’s test did not indicate significant publication bias, the small number of included studies limits the statistical power of this assessment. Additionally, the inability to construct a funnel plot and the potential regional bias from all studies being conducted in China add to the uncertainty. As a precaution, the quality of evidence for all outcomes was downgraded for publication bias.

**TABLE 3:** GRADE evidence profile.

Outcome (no. of studies)	Quality assessment					No. of patients		RR/MD (95% CI)	Certainty	Importance
	Risk of bias	Inconsistency	Indirectness	Imprecision	Publication bias	XYS Combination Therapy	SBT			
ER (6RCTs)	Serious^a^	Not serious	Not serious	Not serious	Serious^b^	311	312	RR = 1.27 (1.18, 1.37)	⊕⊕○○ Low	CRITICAL
FS-14 (6 RCTs)	Serious^a^	Not serious	Not serious	Not serious	Serious^b^	311	312	MD = 1.77 (1.49, 2.06)	⊕⊕○○ Low	CRITICAL
SAS (3 RCTs)	Serious^a^	Not serious	Not serious	Not serious	Serious^b^	170	170	MD = 5.16 (3.84, 6.48)	⊕⊕○○ Low	CRITICAL
SDS (3 RCTs)	Serious^a^	Not serious	Not serious	Not serious	Serious^b^	170	170	MD = 4.62 (3.15, 6.09)	⊕⊕○○ Low	CRITICAL
AEs (4 RCTs)	Serious^a^	Not serious	Not serious	Not serious	Serious^b^	200	200	RR = 0.48 (0.32, 0.72)	⊕⊕○○ Low	CRITICAL

GRADE, Grading of Recommendations, Assessment, Development, and Evaluations; ER, effective rate; FS-14, fatigue scale-14, SAS, self-rating anxiety scale; SDS, self-rating depression scale; RCT, randomized controlled trial; AEs, adverse events.

^a^ The included studies have certain defects in randomization, allocation concealment, and blinding.

^b^ The small number of included studies precluded the construction of a funnel plot to assess the potential for publication bias.

## 4 Discussion

### 4.1 Summary of findings

This study conducted a systematic review and meta-analysis to evaluate the efficacy and safety of XYS in treating CFS. Six RCTs involving 623 patients were included. For the primary outcomes, XYS-based interventions significantly improved the ER compared to SBT alone. Subgroup analyses demonstrated that both XYS alone and the combination of XYS with SBT were effective, with consistent results across studies and low or no heterogeneity. XYS-based interventions also significantly alleviated fatigue symptoms. While moderate heterogeneity was observed overall, subgroup analyses reduced heterogeneity to low levels, likely due to variations in intervention types, dose, dosage form, administration route, and treatment duration of XYS. Sensitivity analysis confirmed the robustness of the findings, demonstrating the stability of the meta-analysis and showing that no single study significantly influenced the pooled results. In terms of secondary outcomes, the combination of XYS and SBT significantly reduced anxiety and depression symptoms compared to SBT alone. Regarding AEs, no adverse reactions were reported when XYS was taken internally alone. Two cases of mild skin redness occurred with the external application of XYS to the navel, likely caused by the stimulating effects of the rhizome of Zingiber officinale Roscoe [Zingiberaceae; Zingiberis rhizoma recens]. When combined with paroxetine, XYS resulted in fewer AEs compared to paroxetine alone. Paroxetine, a commonly used antidepressant, is associated with side effects such as gastrointestinal disturbances (e.g., nausea) and nervous system abnormalities (e.g., dizziness, headache). The AEs observed in the combination group, including dry mouth, nausea, headache, and dizziness, were consistent with the known side effect profile of paroxetine, suggesting that these events were primarily attributable to paroxetine rather than XYS. Previous evidence indicates that XYS, when combined with anxiolytics, can significantly enhance anti-anxiety effects and reduce AEs rates (Wang et al., 2023). Integrating XYS into antidepressant regimens may enhance efficacy while minimizing side effects, offering a promising approach for future clinical applications.

### 4.2 Mechanism of action of XYS on CFS

XYS’s therapeutic effects may stem from its roles in metabolic and immune regulation. Studies have shown that XYS mitigates stress behaviors and balances energy metabolism in CFS models by protecting mitochondrial structure and function, enhancing energy metabolism, and increasing plasma levels of amino acids involved in energy production (Ji et al., 2022; Xu et al., 2022; Zhao W. et al., 2023; Zhao et al., 2024). Immune modulation by XYS includes restoring Th2/Th17 balance, enhancing macrophage cytotoxicity, and improving IgA, IgG, and complement C3 levels while reducing negative immune regulators (Wang et al., 2016; Li et al., 2017; Xu et al., 2023). A clinical trial showed that XYS could significantly increase NK cell activity and IgG and IgM levels in CFS patients, making them close to healthy controls (Shi, 2007). Neuroinflammation, a hallmark of CFS, is addressed by XYS through its neuroprotective effects, reducing oxidative stress, neuroinflammation, and neuronal damage while promoting neuronal recovery (Ji et al., 2021; Liu C. et al., 2021; Zhang et al., 2022b; Su et al., 2023; Lee et al., 2024). Intestinal inflammation, often linked to neuroinflammation in CFS, is ameliorated by XYS, which modulates gut microbiota and reduces inflammation through pathways such as the NLRP3 inflammasome (Komaroff, 2019; Hao et al., 2021; König et al., 2021; Liu et al., 2023b). These mechanisms collectively underpin the observed improvements in fatigue and mental health. Additionally, XYS’s antidepressant and anxiolytic effects are supported by its regulation of the neuroendocrine system. It normalizes hypothalamic-pituitary-adrenal axis activity, modulates neurotransmitter levels, and influences metabolic pathways like tryptophan-kynurenine (Wang et al., 2018; Li et al., 2022; Tong et al., 2023; Wu et al., 2023; Kwon et al., 2024).

### 4.3 Comparison with previous studies

Our findings align with several existing systematic reviews. For instance, Zhang et al. conducted a meta-analysis showing that Chinese herbal medicine relieved fatigue, anxiety, and depression while improving ER (Zhang et al., 2022a). However, the diverse compositions and dosages of Chinese herbal medicine used in the included RCTs contributed to significant clinical heterogeneity. Similarly, Wang et al. found that TCM, whether used alone or in combination with other treatments, significantly reduced fatigue symptoms (Wang et al., 2014). In this review, TCM encompassed a broad range of practices, including herbs, acupuncture, moxibustion, massage, and qigong. Neither of these reviews performed subgroup analyses of specific Chinese herbal formulations.

Furthermore, Hu et al. demonstrated that XYS, either alone or combined with Western medicine, effectively alleviated anxiety in patients with insomnia (Hu et al., 2021). Liu et al. reported that XYS reduced both depression and anxiety in individuals with functional gastrointestinal disorders (Liu Q. et al., 2021), while Jin et al. observed additional benefits of XYS in post-stroke depression (Jin et al., 2018). These findings support our results, highlighting the potential mental health benefits of XYS across various conditions.

### 4.4 Limitations

Several limitations must be acknowledged. First, due to the visual differences between XYS and SBT, none of the included studies implemented double-blinding. Additionally, most studies did not provide sufficient details on randomization, allocation concealment, and blinding of outcome assessments, which may have introduced a high risk of bias. While these factors cannot be changed in the current studies, future research should focus on improving study design by ensuring clear reporting of randomization methods, employing double-dummy placebo-controlled designs, and adhering strictly to blinding protocols to reduce bias. Second, although publication bias was assessed using Egger’s test, the small number of studies included limits the statistical power of this assessment, and thus publication bias cannot be entirely excluded. Future systematic reviews should aim to include a larger body of evidence, ideally incorporating multi-center, high-quality trials to improve the robustness of the findings. Third, variations in treatment durations and follow-up periods across studies, as well as the lack of recurrence rate evaluations, limited the ability to assess the long-term efficacy of XYS. Future studies should standardize the duration of interventions and follow-up periods, and include recurrence rates as an essential outcome to better evaluate the sustainability of the effects. Fourth, the reporting of AEs was inconsistent and lacked standardization across studies. To enhance the reliability of safety assessments, future studies should adopt standardized reporting methods for AEs, evaluate both short- and long-term safety profiles, and consider strategies to minimize any skin irritation caused by external applications of XYS. Fifth, all the studies included in this review were conducted in China, which may introduce regional bias and limit the generalizability of the findings to other populations. Future studies should consider multi-country trials to increase the external validity and broader applicability of the findings. Finally, most studies primarily relied on subjective scales to evaluate CFS symptoms, and lacked objective biomarkers or laboratory indicators, such as immune function, to support the findings. Future research should combine subjective scales with objective measures to provide a more comprehensive and scientifically rigorous assessment of treatment effects.

### 4.5 Strengths and future perspectives

CFS is a debilitating condition that significantly impairs patients’ quality of life. Despite extensive research, effective and safe treatment options remain limited. Our study systematically evaluated the efficacy and safety of XYS in treating CFS, addressing an important gap in the literature. XYS, whether as a standalone therapy or as an adjunct to SBT, significantly improved ER and reduced fatigue in CFS patients. Furthermore, XYS, when combined with the antidepressant, showed potential in alleviating anxiety and depression while reducing AEs. Our findings suggest that XYS could be a valuable alternative treatment option. However, given the low quality of the included studies, future research should focus on addressing the methodological limitations identified in these trials. Rigorous, large-scale RCTs with standardized treatment protocols, comprehensive outcome measures (e.g., biomarkers, immune function), and long-term follow-up are essential to confirm these findings. Incorporating robust designs such as double-dummy placebo-controlled trials will further strengthen the evidence base. By refining study methodologies and exploring the mechanisms underlying XYS’s therapeutic effects, future research can establish its role as a valuable alternative for managing CFS.

## 5 Conclusion

This review indicates that XYS, whether used alone or in combination with SBT, is effective and safe for improving ER, fatigue, anxiety, and depression symptoms in CFS patients. However, clinicians must consider the low quality of evidence and individual patient profiles when integrating XYS into treatment plans. High-quality RCTs with larger sample sizes and longer follow-up are needed to provide stronger evidence for the clinical use of XYS in managing CFS.

## Data Availability

The original contributions presented in the study are included in the article/Supplementary Material, further inquiries can be directed to the corresponding author.

## Abbreviations

CFS, chronic fatigue syndrome; TCM, traditional Chinese medicine; XYS, Xiaoyao San; SBT, standard biomedical treatments; PRISMA, Preferred Reporting Items for Systematic Reviews and Meta-Analyses; RCT, randomized controlled trial; CDC, the Center for Disease Control; ER, effective rate; AEs, adverse events; CI, confidence interval; RR, relative risk; MD, mean difference; GRADE, Grading of Recommendations, Assessment, Development, and Evaluations.

## References

[B1] Araja D. Berkis U. Lunga A. Murovska M. (2021). Shadow burden of undiagnosed myalgic encephalomyelitis/chronic fatigue syndrome (ME/CFS) on society: retrospective and prospective-in light of COVID-19. J. Clin. Med. 10 (14), 3017. 10.3390/jcm10143017 34300183 PMC8303374

[B2] Chang H. Kuo C. F. Yu T. S. Ke L. Y. Hung C. L. Tsai S. Y. (2023). Increased risk of chronic fatigue syndrome following infection: a 17-year population-based cohort study. J. Transl. Med. 21 (1), 804. 10.1186/s12967-023-04636-z 37951920 PMC10638797

[B3] Chen J. Lei C. Li X. Wu Q. Liu C. Ma Q. (2022). Research progress on classical traditional Chinese medicine formula xiaoyaosan in the treatment of depression. Front. Pharmacol. 13, 925514. 10.3389/fphar.2022.925514 35991880 PMC9386002

[B4] Chen Z. (2021). Clinical observation of Xiaoyao powder in treating 33 cases of chronic fatigue syndrome. Clin. J. Chin. Med. 13 (09), 140–141. 10.3969/j.issn.1674-7860.2021.09.052

[B5] Chu L. Valencia I. J. Garvert D. W. Montoya J. G. (2019). Onset patterns and course of myalgic encephalomyelitis/chronic fatigue syndrome. Front. Pediatr. 7, 12. 10.3389/fped.2019.00012 30805319 PMC6370741

[B6] Conroy K. Bhatia S. Islam M. Jason L. A. (2021). Homebound versus bedridden status among those with myalgic encephalomyelitis/chronic fatigue syndrome. Healthc. (Basel) 9 (2), 106. 10.3390/healthcare9020106 PMC7909520 33498489

[B7] Cumpston M. Li T. Page M. J. Chandler J. Welch V. A. Higgins J. P. (2019). Updated guidance for trusted systematic reviews: a new edition of the Cochrane Handbook for Systematic Reviews of Interventions. Cochrane Database Syst. Rev. 10 (10), Ed000142. 10.1002/14651858.Ed000142 31643080 PMC10284251

[B8] Davis H. E. Mccorkell L. Vogel J. M. Topol E. J. (2023). Long COVID: major findings, mechanisms and recommendations. Nat. Rev. Microbiol. 21 (3), 133–146. 10.1038/s41579-022-00846-2 36639608 PMC9839201

[B9] Dukes J. C. Chakan M. Mills A. Marcaurd M. (2021). Approach to fatigue: best practice. Med. Clin. North Am. 105 (1), 137–148. 10.1016/j.mcna.2020.09.007 33246515

[B10] Estévez-López F. Mudie K. Wang-Steverding X. Bakken I. J. Ivanovs A. Castro-Marrero J. (2020). Systematic review of the epidemiological burden of myalgic encephalomyelitis/chronic fatigue syndrome across Europe: current evidence and EUROMENE research recommendations for epidemiology. J. Clin. Med. 9 (5), 1557. 10.3390/jcm9051557 32455633 PMC7290765

[B11] Fukuda K. Straus S. E. Hickie I. Sharpe M. C. Dobbins J. G. Komaroff A. (1994). The chronic fatigue syndrome: a comprehensive approach to its definition and study. International Chronic Fatigue Syndrome Study Group. Ann. Intern Med. 121 (12), 953–959. 10.7326/0003-4819-121-12-199412150-00009 7978722

[B12] Gaunt D. M. Brigden A. Harris S. R. S. Hollingworth W. Jago R. Solomon-Moore E. (2024). Graded exercise therapy compared to activity management for paediatric chronic fatigue syndrome/myalgic encephalomyelitis: pragmatic randomized controlled trial. Eur. J. Pediatr. 183 (5), 2343–2351. 10.1007/s00431-024-05458-x 38429546 PMC11035451

[B13] Grach S. L. Seltzer J. Chon T. Y. Ganesh R. (2023). Diagnosis and management of myalgic encephalomyelitis/chronic fatigue syndrome. Mayo Clin. Proc. 98 (10), 1544–1551. 10.1016/j.mayocp.2023.07.032 37793728

[B14] Guo F. Guo Y. (2015). Clinical research of combined traditional Chinese and western medicine in the treatment of chronic fatigue syndrome. China J. Chin. Med. 30 (01), 133–135. 10.16368/j.issn.1674-8999.2015.01.044

[B15] Ha N. Y. Lee H. Jeong H. Ko S. J. Park J. W. Kim J. (2023). Safety and efficacy of Xiaoyao-san for the treatment of functional dyspepsia: a systematic review and meta-analysis of randomized controlled trials. Front. Pharmacol. 14, 1114222. 10.3389/fphar.2023.1114222 37124216 PMC10130649

[B16] Hao W. Wu J. Yuan N. Gong L. Huang J. Ma Q. (2021). Xiaoyaosan improves antibiotic-induced depressive-like and anxiety-like behavior in mice through modulating the gut microbiota and regulating the NLRP3 inflammasome in the colon. Front. Pharmacol. 12, 619103. 10.3389/fphar.2021.619103 33935710 PMC8087337

[B17] Hu J. Teng J. Wang W. Yang N. Tian H. Zhang W. (2021). Clinical efficacy and safety of traditional Chinese medicine Xiao Yao San in insomnia combined with anxiety. Med. Baltim. 100 (43), e27608. 10.1097/MD.0000000000027608 PMC8556059 34713840

[B18] Ji C. Han Y.-M. Zhao W.-D. Liu X.-Y. Linghu T. Tian J.-S. (2022). Effects of Xiaoyaosan on behavior and skeletal muscle mitochondrial structure and function in depressed rats. Drug Evaluation Research. 45 (09), 1763–1769. 10.7501/j.issn.1674-6376.2022.09.009

[B19] Ji Y. Luo J. Zeng J. Fang Y. Liu R. Luan F. (2021). Xiaoyao pills ameliorate depression-like behaviors and oxidative stress induced by olfactory bulbectomy in rats via the activation of the PIK3CA-AKT1-nfe2l2/BDNF signaling pathway. Front. Pharmacol. 12, 643456. 10.3389/fphar.2021.643456 33935736 PMC8082504

[B20] Jie R. Wang G. (2009). Controlled study of paroxetine combined with Xiaoyao Pills in the treatment of chronic fatigue syndrome. J. Clin. Psychosomatic Dis. 15 (4), 307–308. 10.3969/j.issn.1672-187X.2009.04.0307-03

[B21] Jin X. Jiang M. Gong D. Chen Y. Fan Y. (2018). Efficacy and safety of xiaoyao formula as an adjuvant treatment for post-stroke depression: a meta-analysis. Explore (NY) 14 (3), 224–229. 10.1016/j.explore.2017.12.007 29628336

[B22] Komaroff A. L. (2019). Advances in understanding the pathophysiology of chronic fatigue syndrome. Jama 322 (6), 499–500. 10.1001/jama.2019.8312 31276153

[B23] König R. S. Albrich W. C. Kahlert C. R. Bahr L. S. Löber U. Vernazza P. (2021). The gut microbiome in myalgic encephalomyelitis (ME)/Chronic fatigue syndrome (CFS). Front. Immunol. 12, 628741. 10.3389/fimmu.2021.628741 35046929 PMC8761622

[B24] König R. S. Paris D. H. Sollberger M. Tschopp R. (2024). Identifying the mental health burden in Myalgic Encephalomyelitis/Chronic Fatigue Syndrome (ME/CFS) patients in Switzerland: a pilot study. Heliyon 10 (5), e27031. 10.1016/j.heliyon.2024.e27031 38434357 PMC10907781

[B25] Kuut T. A. Buffart L. M. Braamse A. M. J. Csorba I. Bleijenberg G. Nieuwkerk P. (2024). Does the effect of cognitive behavior therapy for chronic fatigue syndrome (ME/CFS) vary by patient characteristics? A systematic review and individual patient data meta-analysis. Psychol. Med. 54 (3), 447–456. 10.1017/S0033291723003148 37927223

[B26] Kwon T. G. Kim Y. J. Hong J. Y. Song J. H. Park J. Y. (2024). A review of antidepressant and anxiolytic effects of Soyo-san (Xiaoyao-san) and modified Soyo-san in animal models. Phytomedicine 135, 155387. 10.1016/j.phymed.2024.155387 39515106

[B27] Lee J. S. Sato W. Son C. G. (2024). Brain-regional characteristics and neuroinflammation in ME/CFS patients from neuroimaging: a systematic review and meta-analysis. Autoimmun. Rev. 23 (2), 103484. 10.1016/j.autrev.2023.103484 38016575

[B28] Leong K. H. Yip H. T. Kuo C. F. Tsai S. Y. (2022). Treatments of chronic fatigue syndrome and its debilitating comorbidities: a 12-year population-based study. J. Transl. Med. 20 (1), 268. 10.1186/s12967-022-03461-0 35690765 PMC9187893

[B29] Li X. Yu B. Wu X. Xie M. Zeng F. Wei L. (2017). Effects of Xiaoyao Powder on HPA axis and IL-13 and IL-17 in rats with chronic restraint stress syndrome of liver stagnation and spleen deficiency. Lishizhen Med. Materia Medica Res. 28 (08), 1815–1816. 10.3969/j.issn.1008-0805.2017.08.008

[B30] Li Y. Wei D. Zhang M. Yue T. Du H. Liu Q. (2022). Xiaoyao pill improves the affective dysregulation of sleep-deprived female mice by inhibiting brain injury and regulating the content of monoamine neurotransmitter. Curr. Pharm. Biotechnol. 23 (8), 1080–1093. 10.2174/1389201022666211012102501 34636307

[B31] Lim E. J. Ahn Y. C. Jang E. S. Lee S. W. Lee S. H. Son C. G. (2020). Systematic review and meta-analysis of the prevalence of chronic fatigue syndrome/myalgic encephalomyelitis (CFS/ME). J. Transl. Med. 18 (1), 100. 10.1186/s12967-020-02269-0 32093722 PMC7038594

[B32] Liu C. Ying Z. Li Z. Zhang L. Li X. Gong W. (2021a). Danzhi xiaoyao powder promotes neuronal regeneration by downregulating notch signaling pathway in the treatment of generalized anxiety disorder. Front. Pharmacol. 12, 772576. 10.3389/fphar.2021.772576 34912225 PMC8666953

[B33] Liu Q. Shi Z. Zhang T. Jiang T. Luo X. Su X. (2021b). Efficacy and safety of Chinese herbal medicine xiao Yao san in functional gastrointestinal disorders: a meta-analysis and trial sequential analysis of randomized controlled trials. Front. Pharmacol. 12, 821802. 10.3389/fphar.2021.821802 35126152 PMC8811448

[B34] Liu X. Liu S. Ren R. Wang X. Han C. Liu Z. (2023a). A cross-sectional study exploring the relationship between symptoms of anxiety/depression and P50 sensory gating in adult patients diagnosed with chronic fatigue syndrome/myalgic encephalomyelitis. Front. Neurosci. 17, 1286340. 10.3389/fnins.2023.1286340 38249591 PMC10796775

[B35] Liu X. Wu X. Wang S. Qin X. (2023b). Gut microbiome and tissue metabolomics reveal the compatibility effects of Xiaoyaosan on depression based on “gut-liver-kidney” axis. Phytomedicine 111, 154628. 10.1016/j.phymed.2022.154628 36731299

[B36] Luo R. Kuang R. Zhao X. Huang J. (2008). Clinical research guidelines for new traditional Chinese medicines in the treatment of chronic fatigue syndrome. Changsha, Hunan, China, 5.

[B37] Mckeever V. (2024). Long covid and myalgic encephalomyelitis/chronic fatigue syndrome are overlapping conditions. Bmj 384, q613. 10.1136/bmj.q613 38499289

[B38] Qin J. Chen Q. Wang Y. Ge X. Gao A. (2013). Clinical observation on 30 cases of chronic fatigue syndrome treated with Xiaoyao powder applied to the navel. Hebei J. Traditional Chin. Med. 35 (10), 1500–1501. 10.3969/j.issn.1002-2619.2013.10.034

[B39] Salari N. Khodayari Y. Hosseinian-Far A. Zarei H. Rasoulpoor S. Akbari H. (2022). Global prevalence of chronic fatigue syndrome among long COVID-19 patients: a systematic review and meta-analysis. Biopsychosoc. Med. 16 (1), 21. 10.1186/s13030-022-00250-5 36274177 PMC9589726

[B40] Seton K. A. Espejo-Oltra J. A. Giménez-Orenga K. Haagmans R. Ramadan D. J. Mehlsen J. (2024). Advancing research and treatment: an overview of clinical trials in myalgic encephalomyelitis/chronic fatigue syndrome (ME/CFS) and future perspectives. J. Clin. Med. 13 (2), 325. 10.3390/jcm13020325 38256459 PMC10816159

[B41] Shi J. (2019). Analysis of clinical curative effect of modified Xiaoyao san in treatment of chronic fatigue syndrome of liver depression and spleen deficiency. Syst. Med. 4 (01), 135–137. 10.3969/j.issn.2096-1782.2019.01.052

[B42] Shi X. (2007). The efficacy of Xiaoyao Powder in the treatment of chronic fatigue syndrome of liver stagnation and spleen deficiency and its impact on patients’ immune function. Zhejiang J. Traditional Chin. Med. (07), 394–395. 10.3969/j.issn.0411-8421.2007.07.011

[B43] Su P. Wu M. Yin X. Li M. Li Y. Bai M. (2023). Modified Xiaoyao San reverses lipopolysaccharide-induced depression-like behavior through suppressing microglia M1 polarization via enhancing autophagy involved in PI3K/Akt/mTOR pathway in mice. J. Ethnopharmacol. 315, 116659. 10.1016/j.jep.2023.116659 37263314

[B44] Tong Y. Dong L. Shu H. Yang Y. Bai Y. Wen J. (2023). Preclinical evidence evaluation of Xiaoyao san in treating chronic unpredictable mild stress model of depression based on meta-analysis. Phytomedicine 119, 154991. 10.1016/j.phymed.2023.154991 37562092

[B45] Wang J. Li X. He S. Hu L. Guo J. Huang X. (2018). Regulation of the kynurenine metabolism pathway by Xiaoyao San and the underlying effect in the hippocampus of the depressed rat. J. Ethnopharmacol. 214, 13–21. 10.1016/j.jep.2017.11.037 29217494

[B46] Wang Q. Gao S. Zhang W. Zhao Y. He Y. Sun W. (2022). Traditional use and safety evaluation of combination Traditional Chinese Medicine in European registration: with XiaoYao Tablets as an example. Pharmazie 77 (3), 125–130. 10.1691/ph.2022.1227 35459442

[B47] Wang Y. Chen X. Wei W. Ding Y. Guo R. Xing J. (2023). Efficacy and safety of the Chinese herbal medicine Xiao Yao San for treating anxiety: a systematic review with meta-analysis and trial sequential analysis. Front. Pharmacol. 14, 1169292. 10.3389/fphar.2023.1169292 37905203 PMC10613521

[B48] Wang Y. Yu P. Wu J. Hou D. (2016). Effects of xiaoyao powder on cytotoxicity of macrophages in mice with chronic stress. Chin. Archives Traditional Chin. Med. 34 (06), 1297–1299. 10.13193/j.issn.1673-7717.2016.06.004

[B49] Wang Y. Y. Li X. X. Liu J. P. Luo H. Ma L. X. Alraek T. (2014). Traditional Chinese medicine for chronic fatigue syndrome: a systematic review of randomized clinical trials. Complement. Ther. Med. 22 (4), 826–833. 10.1016/j.ctim.2014.06.004 25146086

[B50] Wu Q. Gao J. Bai D. Zhong Y. Yang Z. Jiang X. (2020). Prevalence of chronic fatigue syndrome in China: a meta-analysis. Chin. Youjiang Med. J. 48, 727–735. 10.3969/j.issn.1003-1383.2020.10.002

[B51] Wu W. Z. Ling-Hu T. Zhao Y. H. Zhao W. D. Ji C. Tian J. S. (2023). A unique insight for Xiaoyao San exerts antidepressant effects by modulating hippocampal glucose catabolism using stable isotope-resolved metabolomics. J. Ethnopharmacol. 300, 115702. 10.1016/j.jep.2022.115702 36099982

[B52] Xu W. (2015). Clinical observation on chronic fatigue syndrome treated with integrated traditional Chinese and western medicine. Orient. Diet Ther. Health Care (8), 81.

[B53] Xu X. Xiong L. Ma W. Zhai C. Li W. Tian Y. (2023). Effect of medicinal cake moxibustion on immune proteins and T cell regulatory factors tim-3 and LAG-3 in rats with chronic fatigue syndrome. J. Basic Chin. Med. 29 (10), 1666–1671. 10.19945/j.cnki.issn.1006-3250.2023.10.018

[B54] Xu X. S. Ma W. Xiong L. J. Zhai C. T. Li W. Tian Y. F. (2022). Effect of herbal cake-separated moxibustion on behavioral stress reactions and blood lactic acid level and muscular AMPK/PGC-1α signaling in rats with chronic fatigue syndrome. Zhen Ci Yan Jiu 47 (10), 878–884. 10.13702/j.1000-0607.20220017 36301164

[B55] Zhang Y. Jin F. Wei X. Jin Q. Xie J. Pan Y. (2022a). Chinese herbal medicine for the treatment of chronic fatigue syndrome: a systematic review and meta-analysis. Front. Pharmacol. 13, 958005. 10.3389/fphar.2022.958005 36249791 PMC9557005

[B56] Zhang Y. Luo Y. Hou X. Lu K. He Y. Yang B. (2022b). Xiaoyao powder alleviates the hippocampal neuron damage in chronic unpredictable mild stress-induced depression model rats in hippocampus via connexin 43Cx43/glucocorticoid receptor/brain-derived neurotrophic factor signaling pathway. Bioengineered 13 (1), 383–394. 10.1080/21655979.2021.2005744 34984950 PMC8805874

[B57] Zhao T. Cox I. A. Ahmad H. Campbell J. A. Hensher M. Palmer A. J. (2023a). The economic burden of myalgic encephalomyelitis/chronic fatigue syndrome in Australia. Aust. Health Rev. 47 (6), 707–715. 10.1071/AH23106 38011828

[B58] Zhao W. Han Y. Ji C. Wu W. Zhao Y. Tian J. (2023b). Effects of Xiaoyao San on exercise capacity and liver mitochondria in depressed rats. Drug Eval. Res. 46 (01), 56–63. 10.7501/j.issn.1674-6376.2023.01.008

[B59] Zhao W. Ji C. Zheng J. Zhou S. Tian J. Han Y. (2024). Effects of Xiaoyao San on exercise capacity and liver mitochondrial metabolomics in rat depression model. Chin. Herb. Med. 16 (1), 132–142. 10.1016/j.chmed.2023.09.004 38375048 PMC10874765

