# Supplementary material for: Efficacy and safety of Xiaoyao San in the treatment of chronic fatigue syndrome: a systematic review and meta-analysis
Source: Front Pharmacol. 2025 Feb 6;16:1496774. doi: 10.3389/fphar.2025.1496774 (PMC11839657; doi:10.3389/fphar.2025.1496774)
Supplement: Supplementary file 1 [file DataSheet1.docx]

**Supplemental File**

**Full search strategy of Xiaoyao San for chronic fatigue syndrome**

**PubMed**

#1 “Fatigue Syndrome, Chronic”[Mesh]

#2 Fatigue Syndrome, Chronic[Title/Abstract]

#3 chronic fatigue syndrome[Title/Abstract]

#4 CFS[Title/Abstract]

#5 Chronic Fatigue Disorder[Title/Abstract]

#6 Fatigue Disorder, Chronic[Title/Abstract]

#7 Myalgic Encephalomyelitis[Title/Abstract]

#8 ME [Title/Abstract]

#9 Encephalomyelitis, Myalgic[Title/Abstract]

#10 Systemic Exertion Intolerance Disease[Title/Abstract]

#11 Chronic Fatigue and Immune Dysfunction Syndrome[Title/Abstract]

#12 #1 OR #2 OR #3 OR #4 OR #5 OR #6 OR #7 OR #8 OR #9 OR #10 OR #11

#13 xiaoyaosan [Supplementary Concept]

#14 xiaoyao*[Title/Abstract]

#15 Xiao Yao*[Title/Abstract]

#16 #13 OR #14 OR #15

#17 Randomized controlled trial[Publication Type]

#18 Randomized[Title/Abstract]

#19 placebo[Title/Abstract]

#20 #17 OR #18 OR #19

#21 #12 AND #16 AND #20

**EMBASE**

#1 'chronic fatigue syndrome'/exp

#2 'fatigue syndrome, chronic':ti,ab

#3 'chronic fatigue syndrome':ti,ab

#4 'cfs':ti,ab

#5 'chronic fatigue disorder':ti,ab

#6 'fatigue disorder, chronic':ti,ab

#7 'myalgic encephalomyelitis':ti,ab

#8 'me':ti,ab

#9 'encephalomyelitis, myalgic':ti,ab

#10 'systemic exertion intolerance disease':ti,ab

#11 'chronic fatigue and immune dysfunction syndrome':ti,ab

#12 #1 OR #2 OR #3 OR #4 OR #5 OR #6 OR #7 OR #8 OR #9 OR #10 OR #11

#13 'xiaoyao*':ti,ab

#14 'xiao yao*':ti,ab

#15 #13 OR #14

#16 'random':ti,ab

#17 'placebo':ti,ab

#18 'double-blind':ti,ab

#19 #16 OR #17 OR #18

#20 #12 AND #15 AND #19

**Cochrane Library**

#1 MeSH descriptor: [Fatigue Syndrome, Chronic] explode all trees

#2 (Fatigue Syndrome, Chronic):ti,ab,kw

#3 (chronic fatigue syndrome):ti,ab,kw

#4 (CFS):ti,ab,kw

#5 (Chronic Fatigue Disorder):ti,ab,kw

#6 (Fatigue Disorder, Chronic):ti,ab,kw

#7 (Myalgic Encephalomyelitis):ti,ab,kw

#8 (ME):ti,ab,kw

#9 (Encephalomyelitis, Myalgic):ti,ab,kw

#10 (Systemic Exertion Intolerance Disease):ti,ab,kw

#11 (Chronic Fatigue and Immune Dysfunction Syndrome):ti,ab,kw

#12 #1 OR #2 OR #3 OR #4 OR #5 OR #6 OR #7 OR #8 OR #9 OR #10 OR #11

#13 (xiaoyao*):ti,ab,kw

#14 (Xiao Yao*):ti,ab,kw

#15 #13 OR #14

#16 #12 AND #15

**Web of Science**

#1 TS=(Fatigue Syndrome, Chronic OR chronic fatigue syndrome OR CFS OR Chronic Fatigue Disorder OR Fatigue Disorder, Chronic OR Myalgic Encephalomyelitis OR ME OR Encephalomyelitis, Myalgic OR Systemic Exertion Intolerance Disease OR Chronic Fatigue and Immune Dysfunction Syndrome)

#2 TS=(xiaoyao* OR Xiao Yao*)

#3 TS=(random* controlled trial OR random* OR placebo)

#4 #1 AND #2 AND #3

**Chinese National Knowledge Infrastructure**

(主题:慢性疲劳综合征OR 主题:慢性疲劳综合症 ) AND (主题:逍遥 OR 主题:消遥)

**Chinese Scientific Journals Database**

(U=慢性疲劳综合症 OR U=慢性疲劳综合征) AND (U=逍遥 OR U=消遥)

**Wanfang Data**

(主题:(慢性疲劳综合征) or 主题:(慢性疲劳综合症) ) and (主题:(逍遥) or 主题:(消遥))

**Chinese Biomedicine Literature Database**

#1 "疲劳综合征, 慢性"[不加权:扩展]

#2 慢性疲劳综合征

#3 慢性疲劳综合症

#4 #1 OR #2 OR #3

#5 "逍遥散"[不加权:扩展]

#6 逍遥

#7 消遥

#8 #5 OR #6 OR #7

#9 #4 AND #8
